# Cross neutralization of emerging SARS-CoV-2 variants of concern by antibodies targeting distinct epitopes on spike

**DOI:** 10.21203/rs.3.rs-678247/v1

**Published:** 2021-07-19

**Authors:** Patrick Wilson, Siriruk Changrob, Yanbin Fu, Jenna Guthmiller, Peter Halfmann, Lei Li, Christopher Stamper, Haley Dugan, Molly Accola, William Rehrauer, Nai-Ying Zheng, Min Huang, Jiaolong Wang, Steven Erickson, Henry Utset, Hortencia Graves, Fatima Amanat, D. Noah Sather, Florian Krammer, Yoshihiro Kawaoka

**Affiliations:** University of Chicago; University of Chicago; University of Chicago; University of Chicago; University of Wisconsin - Madison; University of Chicago; University of Chicago; University of Chicago; University of Wisconsin Hospital and Clinics; University of Wisconsin-Madison; University of Chicago; University of Chicago; University of Chicago; University of Chicago; University of Chicago; University of Chicago; Icahn School of Medicine at Mount Sina; Seattle Childrens; Icahn School of Medicine at Mount Sinai; University of Wisconsin-Madison

**Keywords:** SARS-CoV-2, COVID 19, immunity

## Abstract

Several severe acute respiratory syndrome coronavirus 2 (SARS-CoV-2) variants have arisen that exhibit increased viral transmissibility and partial evasion of immunity induced by natural infection and vaccination. To address the specific antibody targets that were affected by recent viral variants, we generated 43 monoclonal antibodies (mAbs) from 10 convalescent donors that bound three distinct domains of the SARS-CoV-2 spike. Viral variants harboring mutations at K417, E484 and N501 could escape most of the highly potent antibodies against the receptor binding domain (RBD). Despite this, we identified 12 neutralizing mAbs against three distinct regions of the spike protein that neutralize SARS-CoV-2 and the variants of concern, including B.1.1.7 (alpha), P.1 (gamma) and B.1.617.2 (delta). Notably, antibodies targeting distinct epitopes could neutralize discrete variants, suggesting different variants may have evolved to disrupt the binding of particular neutralizing antibody classes. These results underscore that humans exposed to wildtype (WT) SARS-CoV-2 do possess neutralizing antibodies against current variants and that it is critical to induce antibodies targeting multiple distinct epitopes of the spike that can neutralize emerging variants of concern.

## Introduction

The emergence of novel circulating SARS-CoV-2 variants of concern (VOCs) have recently proven to undermine the protective effects of infection- and vaccination-induced humoral immunity^1–4^. All approved vaccines against SARS-CoV-2 drive a neutralizing antibody response against the spike protein, the major target of neutralizing antibodies elicited by natural infection^3,5^. However, protective humoral immunity against the spike protein induced by vaccination or infection with the original wildtype (WT) virus may be attenuated due to the widespread circulation of variants^2^. The first reported mutation of the SARS-CoV-2 spike protein, D614G, arose in the C-terminal domain (CTD) and evolved due to increased stability of the spike rather than a mutation to escape host immunity^6^. More recently, mutations have arisen within the receptor-binding domain (RBD), N-terminal domain (NTD) of S1, and S2 that have resulted in emergence of several circulating viral variants that are rapidly becoming the dominant strains around the globe^2^. The B.1.1.7 lineage or alpha VOC, first found in the United Kingdom, has been reported to have a >50% increased transmissibility among humans^7–10^. Of greatest concern is the substitution at position 484 in the RBD, which is exclusively shared by the VOCs and variants of interest (VOIs) originally identified in South Africa (B.1.351; beta), Brazil (P.1; gamma), Texas (R.1), New York (B.1.526; iota) and India (B.1.617.1; kappa)^2,3,11–15^. VOCs possessing a mutation at E484, either E484K and E484Q, can partially evade neutralizing humoral immunity induced by either natural infection or vaccination and, in rare cases, lead to reinfection and infection, respectively^11–13,16–18^. Other emerging variants have acquired a mutation at L452R within the RBD, which is found in B.1.1.298, a variant capable of interspecies transmission between humans and minks, and B.1.427/B.1.429 (epsilon) isolated in southern California^19^.

Moreover, the B.1.617.1 (kappa) found in India possesses both L452R and E484Q mutations within the RBD^15,20^. The most recent VOC, B.1.617.2 (delta), is responsible for a surge in both cases and fatalities in several countries, especially where vaccination rates are low^4,21–23^. Intriguingly, the B.1.617 lineages contain P681R, a mutation that enhances and accelerates viral fusion^24^ and which is also present in the dominant variant in Uganda, A.23.1^25^. Thus, understanding the impact of these various mutations on the neutralization capacity of antibodies elicited by current vaccine formulations or natural exposure to wildtype (WT) SARS-CoV-2 is urgently needed to lay the foundation for next-generation vaccine strategies against SARS-CoV-2 variants.

Here we report that natural WT SARS-CoV-2 infection induces memory B cells expressing potently neutralizing antibodies against VOCs. Moreover, natural WT infection largely induced antibodies against spike epitopes outside of the RBD, most of which were non-neutralizing against WT and VOCs. Additionally, RBD-binding antibodies could be categorized into 3 distinct classes based on their binding profiles against RBD mutant constructs. We identified VOC-neutralizing antibodies against three distinct regions of the spike protein, including the two epitopes on the RBD and one epitope in the NTD. Together, our study identifies that natural WT infection induces memory B cells that can produce neutralizing antibodies against recent SARS-CoV-2 VOCs and have the potential to be recalled by vaccination.

## Results

### Convalescent sera have reduced antibody titers but retain neutralization capabilities against circulating SARS-CoV-2 VOCs

**Figure 1:**
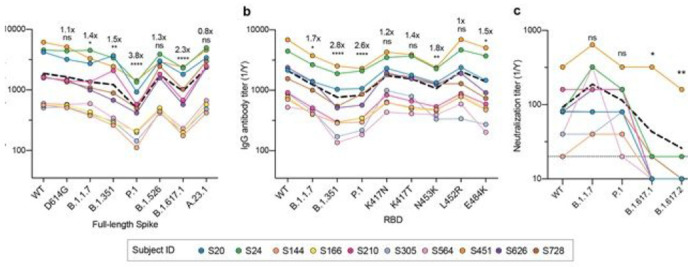
Analyses of serum antibody responses in COVID-19 convalescent individuals. a, b, Total IgG endpoint antibody titers from 10 convalescent subjects against SARS-CoV-2 full-length spike variants (a) and RBD recombinant antigens (b). Dashed line is the mean IgG titer. c, Neutralization titers from 10 convalescent donors against WT SARS-CoV-2, B.1.1.7, P.1, B. 1.617.1 and B. 1.617.2. Dashed line represents the mean neutralization titer. Data in a-c were analyzed using non-parametric Friedman’s test with Dunnett’s multiple comparison test. Fold-change in relative mAb binding to variants or mutants compared to WT in a and b are indicated above the statistical asterisks.

To investigate whether antibodies from subjects naturally infected with WT SARS-CoV-2 lost binding or neutralization activity against VOCs, we collected blood samples from 10 convalescent donors at a median of 49 days post-symptom onset^26,27^ (Supplementary Table 1) for in-depth analysis of specificity of individual memory B cells. As an initial estimate of antibody activity from these patients, serum antibody reactivity was measured comparing reactivity to WT trimeric SARS-CoV-2 spike and spike proteins from the D614G, B.1.1.7, B.1.351, P.1, B.1.526, B.1.617.1 and A.23.1 variants. While serum antibody IgG titers from these 10 patients against WT and D614G spike antigens were similar, titers were significantly reduced against the spike proteins of B.1.1.7 (1.4-fold), B.1.351 (1.5-fold), P.1 (3.8-fold), B.1.526 (1.3-fold), B.1.617.1 (2.3-fold), and A.23.1 (0.8-fold) relative to WT spike protein (Fig. 1a). Similarly, IgG titers against the RBD of B.1.1.7 (1.7-fold), B.1.351 (2.8-fold) and P.1 (2.6-fold) were reduced compared to WT RBD. However, we noted that there was less than a 2-fold decrease in antibody binding against single mutants of the RBD (Fig. 1b). Despite reductions in serum binding activity, the sera retained similar neutralizing titers against the WT, B.1.1.7 and P.1 SARS-CoV-2 variants. However, we found a significant reduction in neutralization against B.1.617.1 and B.1.617.2 compared to WT (Fig. 1c). Although antibody titers were lower against the VOCs, these data indicate that serum antibodies elicited by natural WT infection were able to neutralize B.1.1.7, P.1 and WT virus equally, while most donors lost neutralizing potential against B.1.617-lineage viruses.

### Generation of mAbs against distinct domains of the SARS-CoV-2 spike

**Figure 2:**
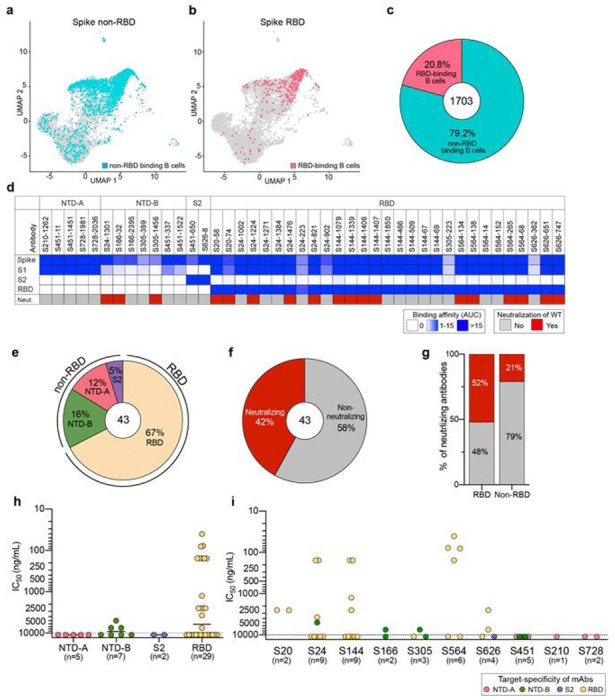
Characterization of spike-reactive mAbs. a, b, Uniform manifold approximation and projection (UMAP) of SARS-CoV-2 spike non-RBD binding (a) and spike RBD binding B cells (b) isolated from the PBMCs of 10 convalescent subjects. c, The proportion of spike non-RBD and spike RBD specific binding B cells. The number in center of pie chart indicates the number of antigen-specific binding B cells. d, mAbs generated from selected B cells (n=43) were tested for binding to full-length spike, S1, S2, and RBD and neutralization potential against WT SARS-CoV-2. Binding data are represented as area under the curve (AUC). Neutralizing activity less than 10,000 ng/ml are considered neutralizing. e, f, Pie charts of mAbs domain specificity (e) and neutralizing capability (f). Number in the center of pie graphs indicate the number of antibodies tested. g, Comparison of neutralizing capability of mAbs targeting spike RBD and spike non-RBD. h and i, IC50 of neutralization potency of spike-reactive antibodies against WT virus based on domain specificity (h) and by subject (i). Mean in h indicated as a solid line. Data in h and i are colored based on domain specificity and dashed lines shown in h and i indicate limit of detection (10,000 ng/mL). Data in d-i are representative of two independent experiment performed in duplicate. Genetic characterization of each mAb is further detailed in Extended Data Table 2.

We next sought to determine the specificities of antibodies that could cross-neutralize these viral variants by generating mAbs from spike-binding B cells isolated from 10 convalescent subjects collected between April and July of 2020^26,27^. We sort-purified B cells binding to spike and/or RBD fluorophore- and oligo-conjugated probes, and performed single-cell RNA-sequencing and B cell receptor sequencing. As the antigen probes included a DNA oligonucleotide sequence, we were able to track the antigen-specificity of isolated B cells. In total, we obtained 1,703 paired immunoglobulin heavy and light chains from non-RBD- and RBD-binding B cells specific for the spike. Overall, the percentage of spike non-RBD-binding B cells was 4-fold higher than RBD-binding B cells (Fig. 2a–c), indicating that natural WT infection preferentially induced B cell response toward epitopes on the spike outside of the RBD^28,29^. Overall, B cells targeting the RBD or epitopes outside of the RBD utilized similar V(D)J genes, had overlapping heavy and light chain pairings, and possessed similar numbers of mutations and complementarity determining region 3 (CDR3) lengths (Supplementary Figure 1a–i).

Based on the acquired antibody sequences and probe-binding intensities, we generated 43 mAbs from all 10 donors specific to the WT spike protein (Supplementary Table 2). To investigate specific domain targeting, mAbs were tested for binding to the RBD and monomeric S1 and S2 recombinant spike antigens. Based on binding to these discrete antigens, spike-reactive mAbs were categorized into 4 groups: NTD-A-reactive mAbs (n=5) that bound strongly to S1 but not RBD, NTD-B-reactive mAbs (n=7) that weakly bind S1 but not RBD, S2-reactive mAbs (n=2), and RBD-reactive mAbs (n=29) (Fig. 2d–e). Additionally, NTD-A and NTD-B-classified antibodies targeted distinct epitopes as shown by competition ELISA (Supplementary Figure 2a). We further determined whether antibodies with different binding specificities differ in their neutralization capacity against WT SARS-CoV-2. Of the 43 mAbs, 18 (42%) were neutralizing. Notably, only mAbs binding the RBD and NTD-B were neutralizing, whereas all mAbs binding NTD-A and S2 were non-neutralizing (Fig. 2d–f). Moreover, 52% of RBD-targeting mAbs were neutralizing, with eight mAbs being potently neutralizing antibodies (50% inhibitory concentration, IC_50_, of < 500 ng/ml), and three out of seven NTD-B mAbs having moderate neutralization potency (5,000-7,500 ng/ml) (Fig. 2g–h). Of the 10 convalescent donors, seven had at least one neutralizing mAb among the antibodies cloned for this study, although the potencies of the mAbs varied by donor (Fig. 2i). Together, these data reveal that mAbs against the RBD are the predominate source of neutralizing antibodies induced by WT SARS-CoV-2 infection.

### Binding and neutralizing breadth of non-RBD spike antibodies

**Figure 3:**
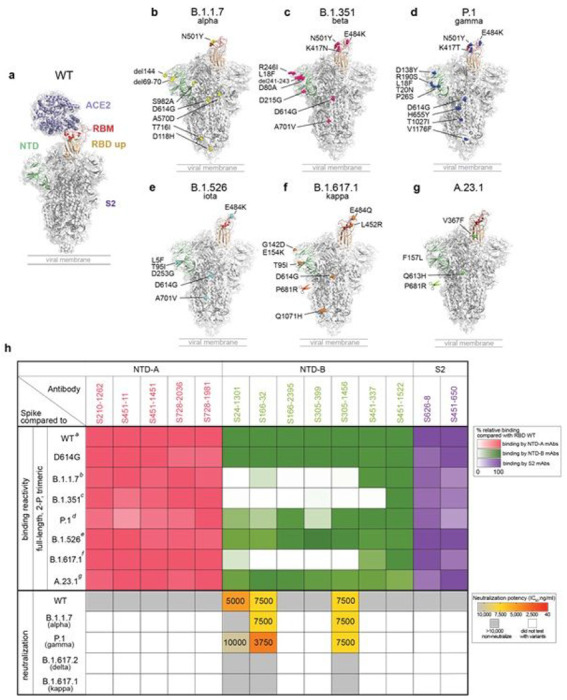
Binding breadth and neutralization of spike non-RBD mAbs. a, Full-length spike protein binding to ACE2 (a; PDB: 7KJ2). b-g, Locations of mutations found on B.1.1.7 (b), B.1.351 (c), P.1 (d), B.1.526 (e), B.1.617.1 (f) and A.23.1 (g). (b-g; modified from PDB: 6XM4). h, The binding reactivity and neutralization capabilities of NTA-A (pink), NTD-B (green) and S2 reactive mAbs (purple). The color gradients indicate percentage of relative binding compared to WT spike. The neutralization potency (IC50) of spike-non RBD mAbs against WT, B.1.1.7, P.1, B.1.617.1 and B.1.617.2 variants are indicated as ng/ml. The panel of SARS-CoV-2 viruses are detailed in Extended Data Table 4. Data in h are representative of two independent experiments performed in duplicate. Genetic information for each mAb is in Extended Data Table 2.

To understand the effects of viral variants on mAb binding to epitopes on the spike outside of the RBD, we tested non-RBD-targeting mAbs for binding to a panel of SARS-CoV-2 variants, including D614G and the emerging variants B.1.1.7, B.1.351, P.1, B.1.526, B.1.617.1 and A.23.1 (Fig. 3a–g). All non-RBD spike-reactive antibodies showed similar binding to the D614G spike. Furthermore, all mAbs targeting NTD-A and S2 maintained similar binding to the spike of the B.1.1.7, B.1.351, P.1, B.1.526, B.1.617.1 and A.23.1 variants (Fig. 3h). Although mAbs against NTD-A and S2 retain binding to VOCs, they are non-neutralizing, implying that NTD-A- and S2-reactive antibodies may provide limited immune pressure to mutate these epitopes. Of interest, NTD-B mAbs showed significantly reduced binding to the spike of B.1.1.7, B.1.351 and B.1.617.1 while showing similar binding to B.1.526 and A.23.1, and a minor reduction in binding to the spike of P.1 (Fig. 3h). Two of the three neutralizing NTD-B binding mAbs (S166-32 and S305-1456), which were isolated from two different subjects, retained neutralization potential against B.1.1.7 and P.1 at moderate neutralizing potency (Fig. 3h). The third neutralizing NTD-B-binding mAb (S24-1301) also had moderate neutralizing potency against the WT strain with weak cross-neutralization activity against the P.1 variant and no neutralization activity against B.1.1.7, consistent with its binding profile (Fig. 3h). However, all three neutralizing NTD-B mAbs failed to neutralize B.1.617.1 and B.1.617.2. Together, our data indicate that antibodies against NTD-B show cross-neutralization capacity and thus may provide protection against some emerging VOCs, such as B.1.1.7 and P.1. However, antibodies targeting the NTD-B epitope may be driving spike evolution, particularly the mutations and deletions found within B.1.1.7, B.1.351, B.1.617.1 and B.1.617.2, leaving the future of this epitope as a reliable target for cross-reactive antibodies uncertain.

### A subset of RBD-binding mAbs retain neutralization activity against VOCs

**Figure 4:**
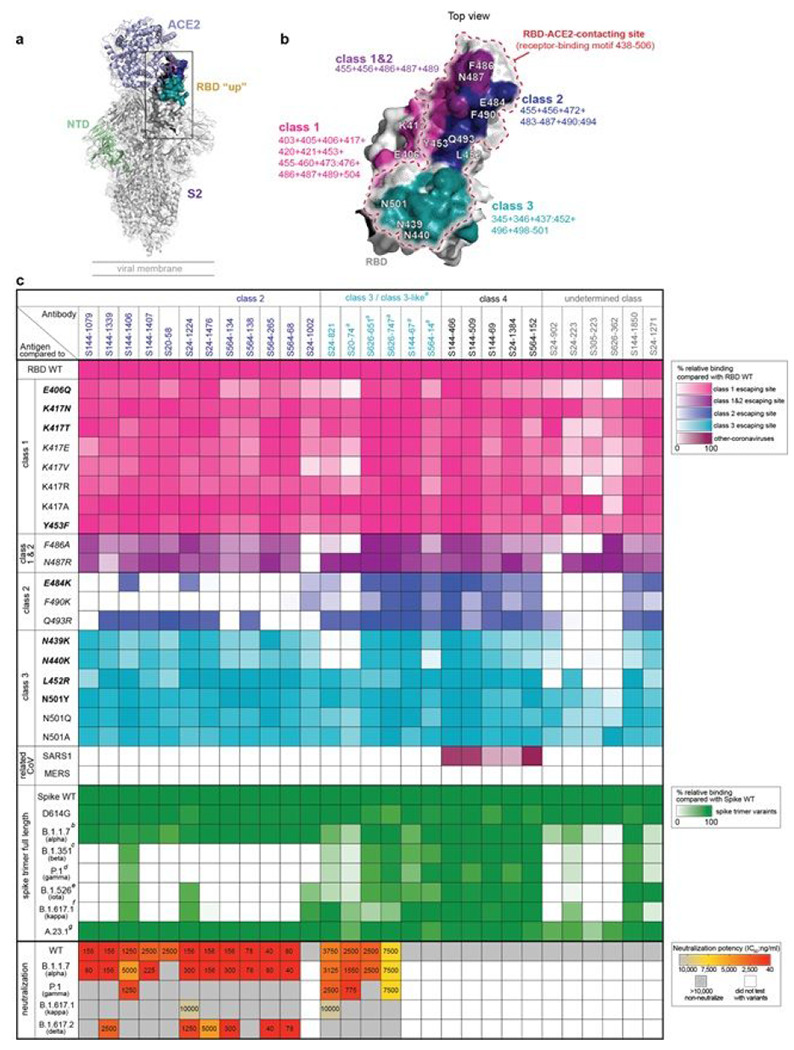
Binding and neutralization profiles of RBD-binding mAbs against a panel of RBD escape mutants and variants. a, Structural model of RBD “up” binding with ACE2 (a; PDB: 7KJ2) and RBD antibody classes and associated escape mutants. b, RBD is colored by antibody classes and associated mutations: class 1 (pink), overlapping of class 1 and 2 (purple), class 2 (blue), class 3 (teal). c, Heatmap detailing binding reactivity of RBD mAbs (n=29) against single key escape sites for class 1, class 2 and class 3 antibodies, combinations of RBD mutants, and RBD from SARS-CoV-1 and MERS-CoV. Abbreviation of a refers to class-3 like antibodies, which are defined by mAbs that compete with a class 3 mAb (Extended Data Fig. 2c). Abbreviations b-f refer to mutations in the RBD of each full length spike variant, B.1.1.7 with N501Y (b), B.1.351 with K417N:E484K:N501Y (c), P.1 with K417T:E484K:N501Y (d), B.1.526 with E484K (e), B.1.617.1 with L452R:E484Q (f) and A.23.1 with V367F (g). The panel of recombinant antigens in c are detailed in Extended Data Table 3, including mutations found in circulating SARS-CoV-2 variants (bold), the mutations that escape/reduce binding by polyclonal serum/potent neutralizing mAbs (italic), the mutations found in both circulating SARS-CoV-2 variants and in vitro escape-map (bold+italic), and artificial mutants at key contact residues of the RBD-ACE2 interaction (normal typeface). The neutralization potency (IC50) of spike-RBD mAbs against WT, B.1.1.7, P.1, B.1.617.1 and B.1.617.2 variants are indicated as ng/ml. The panel of SARS-CoV-2 viruses are detailed in Extended Data Table 4. Data in c are representative of two independent experiments performed in duplicate. Genetic information for each antibody is in Extended Data Table 2.

Viral escape mutations occurring within the RBD may result in reduction in neutralization capacity of RBD-targeting antibodies^30–32^. To understand the impacts of RBD mutations on mAb binding, we tested RBD-targeting mAbs for binding to RBD mutants that possessed a single mutation found in circulating SARS-CoV-2 VOCs, VOIs, or artificial mutants at key contact residues of the RBD-ACE2 interaction^30–35^, as well as full-length spike constructs containing multiple mutations in the RBD (Supplementary Table 3). In addition, we tested mAb binding to the RBDs of SARS-CoV-1 and Middle Eastern Respiratory Syndrome (MERS)-CoV to investigate cross-reactivity to other coronaviruses. Notably, RBD-binding mAbs have been classified into four classes, classes 1–4 or receptor binding site (RBS) A-D, based on structural analysis and antibody binding features^36,37^. More recently, classification of four key antigenic regions of the RBD can also be defined by determining the loss of binding to RBD mutants (class 1–3 epitopes) or binding to cryptic epitopes on the RBD that are conserved across SARS-CoV-1 and MERS-CoV RBDs (class 4 epitope, Fig. 4a–b)^30,38^. Based on the binding profiles of class 1-4 binding mAbs, we were able to segregate 23 out of 29 mAbs into one of the four classes (Fig. 4c and Supplementary Figure 2b). Notably, no class 1 mAbs were found and six mAbs could not be classified as they either lost binding to multiple mutant classes or bound equally to all RBD mutants but did not bind to SARS-CoV-1 or MERS-CoV.

Class 2 RBD-binding mAbs showed reduced binding to at least one of the RBD class 2 single escape mutants, notably E484K and F490K, and the majority of these mAbs lost binding to the RBD mutants found in the B.1.351, P.1, B.1.526 and B.1.617.1 (Fig. 4c). Of the 12 class 2 mAbs, 11 were potently neutralizing against WT SARS-CoV-2. Of the neutralizing class 2 mAbs, all but one neutralized B.1.1.7 at concentrations comparable to neutralization of the WT strain. By contrast, six neutralized B.1.617.2 at lower potency compared to WT and B.1.1.7. Seven of the class 2 mAbs retained their neutralization activity against at least two VOCs (Fig. 4c). Of note, 10 out of 11 neutralizing class 2 mAbs were unable to neutralize the variants that harbored a mutation at E484, P.1 and B.1.617.1. This is in line with previous studies, which have shown that the E484K and E484Q mutations are the key escaping residue responsible for neutralization resistance by P.1, P.2, B.1.351 and B.1.617.1 VOCs^2,4,39^. Of greatest interest, S144-1406, which retained binding to E484K and to all spike variants, neutralized B.1.1.7 and P.1 variants with high neutralization potency. Similar to another E484K-binder, S24-1224 neutralized three out of four VOCs tested, including B.1.617.1 (Fig. 4c). These data indicate that some class 2 antibodies can cross-neutralize VOCs. Additionally, the epitope targeted by S144-1406 partially overlapped with S24-1224 and other class 2 mAbs that failed to neutralize P.1 and B.1.617.1 (Supplementary Figure 2c), suggesting class 2 mAbs target similar but slightly different RBD epitopes.

Only one mAb (S24-821) specifically lost binding to the class 3 mutants, particularly to N439K and N440K, which are associated with circulating VOCs^35,40^ and have been reported as *in vitro* escape sites for class 3 epitope-binding mAbs^30,31,35^ (Fig. 4b and Supplementary Table 3). Moreover, we classified five more mAbs as class 3-like as they strongly competed for RBD binding with S24-821 but did not compete with class 2 mAbs (Supplementary Figure 2b). Importantly, all class 3 and class 3-like mAbs maintained binding to L452R, another mutation associated with class 3 antibodies that is present in B.1.427/B.1.429^19,41^ and B.1.617 variants^20^ (Fig. 4c). Of the four neutralizing class 3 and class 3-like mAbs, all four retained neutralization activity against B.1.1.7 and three were neutralizing against P.1 (Fig. 4b). In contrast to class 2 mAbs, B.1.617.2 was resistant to all class 3-neutralizing mAbs. Only one mAb (S24-821) retained modest neutralization potency to B.1.617.1, indicating antibodies binding class 3 epitopes could neutralize some VOCs even though they bound L452R single mutation and all spike variants.

All of the mAbs that were categorized into class 4 (n=5) maintained binding to all RBD mutants and spike variants and displayed cross-reactivity to the SARS-CoV-1 RBD. However, all class 4 mAbs were non-neutralizing against WT virus, suggesting antibodies against this epitope are likely not strong drivers of antigenic drift. Notably, three antibodies in the class 4 group utilized the same heavy chain gene, VH5-51, as CR3022 and competed with CR3022 for binding to the RBD, indicating the class 4 antibodies in our study likely target the same or a similar epitope as CR3022 (Supplementary Table 2 and Supplementary Figure 2d). This is consistent with a previous study showing CR3022 cross-reacts with SARS-CoV-1, suggesting class 4 antibodies are common across subjects and studies^17,30^.

With the classification of mAbs against distinct epitopes, we next tested the relative abundance of serum antibodies against these distinct epitopes of the RBD and NTD by performing competition assays. Notably, donors had significantly higher titers of serum antibodies targeting class 3 (S24-821) and class 3-like (S20-74) epitopes, whereas subjects largely had undetectable titers against class 2 and NTD-B epitopes, suggesting WT SARS-CoV-2 infection predominately induces polyclonal antibodies targeting RBD class 3 epitopes that can neutralize emerging VOCs B.1.1.7 and P.1. (Supplementary Figure 2e). These data are consistent with the observed anti-B.1.1.7 and anti-P.1 serum neutralizing titers shown in Fig. 1c, suggesting the retention of serum neutralization activity could be due to abundant class 3 antibody responses. Loss of neutralization capabilities to B.1.617-lineage viruses may be due to insufficient levels of class 2 serum antibodies. A comparison of the neutralization capabilities of mAbs targeting different epitopes revealed class 2 RBD-reactive mAbs were the most potently neutralizing followed by mAbs targeting class 3 RBD epitopes and NTD-B (Supplementary Figure 3a). It is important to note that none of neutralizing mAbs induced by natural WT infection were able to neutralize all emerging SARS-CoV-2 variants. Nonetheless, we identified at least one mAb that could neutralize each VOC, suggesting the convalescent donors generated a diverse cross-neutralizing antibody response (Supplementary Figure 3b). Therefore, antibodies targeting multiple epitopes on the spike are a valuable source of neutralizing antibodies against emerging VOCs. Moreover, the cross-neutralizing RBD-targeting mAbs used V(D)J gene features similar to other published RBD-binding mAbs (Supplementary Table 3)^42–44^. However, the mAbs in our studies utilized distinct heavy and light chain pairings, indicating these clones are not public with other known neutralizing SARS-CoV-2 antibodies. Despite this, our data indicate that cross-neutralizing antibodies use a diverse antibody repertoire against multiple distinct epitopes. Therefore, driving a polyclonal antibody response against these three epitopes may provide cross-neutralizing protection against existing and future variants.

## Discussion

Our study shows WT SARS-CoV-2-convalescent individuals possess antibodies that can effectively cross-neutralize against emerging VOCs, with cross-neutralizing antibodies targeting multiple epitopes of the spike protein. In total, we identified 12 mAbs that potently neutralize current circulating VOCs, including B.1.1.7, the alpha variant that has been reported to be more infectious^8,19^, P.1, the gamma variant that partially escapes both natural and vaccine-induced humoral immunity^2,12,45^, and B.1.617.2, the delta variant that is more transmissible than the alpha variant and has led to a surge of more hospitalizations in India and can evade partial immunity induced by one vaccine dose^4,15,23^. Convalescent subjects in our cohort had sufficient serum titers to neutralize both B.1.1.7 and P.1 but not B.1.617, suggesting that the cross-neutralizing mAbs identified in this study may play an important role in polyclonal neutralization for some of VOCs.

Using high-throughput antigen probing at the single B cell level, we found that B cells isolated from convalescent subjects largely targeted non-RBD epitopes rather than potently neutralizing epitopes on the RBD. Similarly, mRNA vaccines also largely induce antibodies against non-neutralizing epitopes, suggesting epitopes outside of the RBD are immunodominant^46^. Despite this, vaccination has been shown to induce cross-neutralizing antibodies^1^, suggesting both natural WT infection and currently approved vaccines can elicit protective humoral immunity against emerging variants. As we identified 12 antibodies cross-neutralizing to VOCs derived from seven different convalescent COVID-19 donors, our study suggests most people generate a cross-neutralizing antibody response. Notably, these antibodies largely target three distinct epitopes, including two sites on the RBD and the one on the NTD. Several recent studies have demonstrated that antibodies against the NTD and S2 are neutralizing^47–49^. Although the anti-S2 mAbs identified in our study were non-neutralizing, S2-binding antibodies exhibit broad reactivity with spike proteins from SARS-CoV-2 variants, related betacoronaviruses such as SARS-CoV-1 and MERS-CoV, and distantly related endemic coronaviruses. Moreover, anti-spike serum antibodies can mediate protection via Fc-mediated functions, suggesting a combination of neutralizing antibodies and polyfunctional antibodies will provide optimal protection against infection with variants of SARS-CoV-2^50^.

Our study also showed that anti-RBD mAbs are primarily class 2 mAbs, consistent with other reports^30,37,43,51^. The majority of class 2 mAbs retained their neutralization activity against B.1.1.7 and B.1.617.2, but were largely non-neutralizing against P.1, suggesting class 2 mAbs may have driven the evolution of P.1 mutants. In contrast, neutralizing class 3 mAbs retained their neutralization activity against both B.1.1.7 and P.1, but did not neutralize the B.1.617 variants. Notably, none of the neutralizing mAbs could cross-neutralize B.1.1.7, P.1 and B.1.617.2, the most prevalent VOCs at this time. Therefore, vaccination approaches to increase affinity and frequencies of antibodies to the S1 domain may enhance the breadth of protection against emerging SARS-CoV-2 VOCs, including epitopes on the RBD and NTD. It is likely that targeting multiple epitopes will provide optimal protection so as to avoid generating escape mutants that can evade antibodies against any one epitope. Moreover, vaccinating previously infected subjects has been shown to substantially improve neutralization titers^3^ and may allow for refinement of memory B cells against neutralizing epitopes.

In conclusion, our study shows SARS-CoV-2 infection induces cross-neutralizing immunity against circulating VOCs, which is likely attributed to polyclonal antibodies targeting multiple epitopes of the spike protein. This work emphasizes the need for the induction of cross-neutralizing antibodies that bind distinct sites on the spike with various mechanisms that can synergize to provide protection against SARS-CoV-2 variants as well as limit the virus from escaping any single antibody target.

## Materials And Methods

### Study cohort and spike-specific B cells sorting

All studies were performed with the approval of the University of Chicago institutional review board IRB20-0523 and University of Chicago, University of Wisconsin-Madison. Informed consent was obtained after the research applications and possible consequences of the studies were disclosed to study subjects. This clinical trial was registered at ClinicalTrials.gov with identifier NCT04340050, and clinical information for patients included in the study is detailed in **Extended Data Table 1**. The details of PBMC collection from leukoreduction filters were described elsewhere^27^. For spike-specific B cells sorting, PBMC were thawed in 37°C water bath and B cells were enriched using human pan B cell EasySepTM enrichment kit (STEMCELL). B cells were stained with anti-CD19-PE-Cy7 (Biolegend) and anti-CD3-BV510 (BD Biosciences) and antigen probes (PE) for 30 minutes on ice in 1X PBS supplemented with 0.2% BSA and 2 mM Pierce Biotin. Probe generation was performed as previously described^27^. Cells were subsequently washed with 1X PBS with 0.2% BSA and stained with Live/Dead BV510 (Thermo Fisher) in 1X PBS for 15 minutes. Cells were washed again and re-suspended at a maximum of 4 million cells/mL in 1X PBS supplemented with 0.2% BSA and 2 mM Pierce Biotin for downstream cell sorting using the MACSQuantTyto cartridge sorting platform (Miltenyi). Viable/CD19+/antigen-PE+ cells were sorted as probe positive. Cells were then collected from the cartridge sorting chamber and used for downstream processing with the chromium controller (10X Genomics).

### Single-cell RNA-seq and B cell receptor sequencing

The human B cell V(D)J, 5’ gene expression, feature barcode libraries were prepared according to manufacturer’s instructions. Libraries were pooled and sequenced using an Illumina NextSeq550 or an Illumina NextSeq 500 at the University of Chicago. Cell Ranger (version 3.0.2) was used to perform raw sequencing processing, sample de-multiplexing, barcode processing, single-cell 5’ transcripts counting and B cell receptor repertoire sequences assembly. The reference genome assembly for transcriptome is GRCh38-1.2.0, and reference genome assembly for V(D)J is cellranger-vdj-GRCh38-alts-ensembl-2.0.0. The data obtained from Cell Ranger were subsequently performed downstream analysis using Seurat toolkit (version 3.2.0, an R package, for transcriptome, cell surface protein and antigen probe analysis)^52^ and IgBlast (version 1.15) for immunoglobulin gene analysis^53^. Cell quality control (QC), normalization, data scaling, and linear dimensional reduction, clustering, differential expression analysis, batch effects correction, and data visualization were processed using Seurat (version 3.2.0). The QC of cells were performed further to exclude cells with less than 200 and more then 2500 detected genes and cells expressing high percentage of mitochondrial genes. Transcriptome RNA data was analyzed using conventional log normalization. We performed principal component analysis (PCA) and used the top 15 principal components (PCs) in linear dimensional reduction and clustering. Only filtered, high-quality cells were clustered in this analysis using Louvain algorithm implemented in Seurat under the resolution of 0.6 for clustering. Batch effects across different datasets were normalized using an Anchor method implemented in Seurat.

### Monoclonal antibody production

B cells were selected for mAb generation based on antigen probe intensity visualized by JMP Pro 15, as previous described^27^. Antibody heavy and light chain genes obtained by 10X Genomics V(D)J sequencing analysis were synthesized by Integrated DNA Technologies. The synthesized fragments for heavy and light chain with 5’ and 3’ Gibson overhangs were then cloned into human IgG1 and human kappa or lambda light chain expression vectors by Gibson assembly as previously described^54^. The heavy and light chains of a corresponding mAb were co-transfected into HEK293T cells. After 4 days, secreted mAbs in the medium supernatant were harvested and purified using protein A agarose beads (Thermo Fisher).

### Recombinant proteins

The recombinant WT SARS-CoV-2 full-length (FL) spike, D614G FL spike, WT RBD, K417T/R/A RBD, N501Q/A RBD, and SARS-CoV-1 RBD and MERS-CoV were generated in-house either by using gBlock fragment synthesized by Integrated DNA Technologies or by performing single-site mutagenesis, and expressed by Expi293F cells (Thermo Fisher). The recombinant FL spikes derived from variants of B.1.1.7, B.1.351, P.1, B.1.526, B.1.617.1 and A.23.1 were kindly provided by Dr. Noah Sather laboratory at Seattle Children’s Research Institute. The recombinant RBD found in VOCs, B.1.351 or P.1 variants, and RBD with single mutation or multiple mutations (N439:Y453F, E406Q, K417E, K417V, Y453F, F486A, N487R, F490K, Q493R, N439K, N440K, N501Y) were generously provided from the Krammer laboratory at Icahn School of Medicine at Mount Sinai. The recombinant S1 and S2 subunit, and RBD with single mutation of K417N, E484K and L452R were obtained from Sino Biological. The protein sequences and resources for each antigen are listed in **Extended Data Table 3**.

### Virus neutralization assay

Virus neutralization assays were performed with different variants of SARS-CoV-2 on Vero E6/TMPRSS2 (**Extended Data Table 4**). Virus (~100 plaque-forming units) was incubated with an equal volume of twofold diluted of serum or mAbs for 1 hour. Plasma samples were diluted in calcium free media, while antibodies were diluted in growth media. In addition, plasma was heat treated for 30 minutes at 37 °C prior to use. The antibody/virus mixture was added to confluent Vero E6/TMPRSS2 cells that were plated at 30,000 cells per well the day prior in 96-well plates. The cells were incubated for 3 days at 37 °C and then fixed and stained with 20% methanol and crystal violet solution. Virus neutralization titers were determined as the reciprocal of the highest serum dilution that completely prevented cytopathic effects. The 50% inhibitory concentrations for mAbs (IC50) was determined using log(inhibitor) versus normalized response (variable slope) performed by Prism (Graphpad Version 9.0). All plasma and mAbs were tested in duplicate and each experiment was performed twice.

### Enzyme-linked immunosorbent assay (ELISA)

High-protein binding microtiter plates (Costar) were coated with 50 μl of recombinant proteins (either full-length spike or RBD) at 2 μg/ml in 1×PBS solution overnight at 4°C. The plates were washed 3 times the next day with 1×PBS supplemented with 0.05% Tween 20 and blocked with 175 μl of 1×PBS containing 20% FBS for 1 hour at 37°C. MAbs were serially diluted 1:3 starting at 10 μg/ml and incubated for 1 hour at 37°C. The plates were then washed 3 times and incubated with horseradish peroxidase (HRP)-conjugated goat anti-human IgG antibody (Jackson ImmunoResearch) diluted 1:1000 for 1 hour at 37°C, and plates were subsequently developed with Super AquaBlue ELISA substrate (eBioscience). Absorbance was measured at 405 nm on a microplate spectrophotometer (Bio-Rad). To standardize the assays, control antibodies with known binding characteristics were included on each plate and the plates were developed when the absorbance of the control reached 3.0 OD405 units. All mAbs were tested in duplicate and each experiment was performed twice.

### Competition ELISAs

To determine the classification of certain mAbs, competition ELISAs were carried out using the mAbs with known epitope binding property as competitor mAbs. The competitor mAbs were biotinylated overnight at 4°C with EZ-LinkÔ Sulfo-NHS-Biotin (Thermo Scientific). The excess free biotin of biotinylated mAbs were removed by 7k MWCO ZebaÔ spin desalted columns (Thermo Scientific). Plates were coated with 50 μl of 2 μg/ml RBD antigen overnight at 4°C. After 1 hour of blocking the plates with PBS 20% FBS, the 2-fold dilution of undetermined class mAbs or serum were added (starting at 20 μg/ml of mAbs and 1:50 of serum) into coated well. After incubated for 2 hours at room temperature, biotinylated competitor mAb was added at a concentration of 2x K_d_ and incubated another 2 hours at room temperature together with mAbs or serum that were previously added. The plates were washed 3 times and incubated with 100 μl HRP-conjugated streptavidin (Southern Biotech) dilution of 1:1000 for 1 hour at 37°C. The plates were developed with Super AquaBlue ELISA substrate (eBioscience). To standardize the assays, competitor biotinylated mAb was added in well that without any competing mAbs or serum as control well. The data were recorded when the absorbance of the control well reached 1 to 1.5 OD405 units. All mAbs were tested in duplicate and each experiment was performed twice. The percent competition was then calculated by dividing a sample’s observe OD by the OD reached by the positive control, subtracting this value from 1, and multiplying by 100. For the serum data, ODs were log transformed and analyzed by non-linear regression to determine EC50 values using Prism software (Graphpad Version 9.0).

### Biolayer interferometry (BLI)

To determine the classification of certain mAbs, competition assays were performed using the mAbs with known epitope binding property as competitor mAbs with mAb binding unknown epitopes using BLI with a Octet K2 instrument (Forte Bio). The RBD of SARS-CoV-2 was biotinylated, desalted and loaded at a concentration of 10 μg/ml onto streptavidin probes for 300 seconds followed by PBS for 60 seconds. The probe was moved to associate with mAbs of interest (10 μg/ml) for 300 seconds followed by PBS for 60 seconds and then associations with control mAbs (10 μg/mL) for 300 seconds. The final volume for all the solutions was 200 ml/well. All of the assays were performed with PBS buffer at 30°C.

### SARS-CoV-2 Spike and RBD protein models

FL mutations were visualized on the WT spike protein (PDB: 7KJ2) using PyMOL (Schrödinger). The model of RBD mutations and RBD classes were visualized on the WT RBD protein (PDB: 7KDL) using PyMOL (Schrödinger). The models were further processed by Adobe Illustrator 2021 and Adobe Photoshop.

### Statistical analysis

All statistical analyses were performed using Prism software (Graphpad Version 9.0). Sample sizes (n) for the number of mAbs tested are indicated in corresponding figures or in the center of pie graphs. Number of biological repeats for experiments and specific tests for statistical significance used are indicated in the corresponding figure legends. *P* values less than or equal to 0.05 were considered significant. * *P* ≤ 0.05, ** *P* ≤ 0.01, *** *P* ≤ 0.001, **** *P* < 0.0001.

## Supplementary Material

Supplement 1

Supplement 2
