# Supplementary material for: Cross neutralization of emerging SARS-CoV-2 variants of concern by antibodies targeting distinct epitopes on spike
Source: Res Sq. 2021 Jul 19:rs.3.rs-678247. Preprint. [Version 1] doi: 10.21203/rs.3.rs-678247/v1 (PMC8312900; doi:10.21203/rs.3.rs-678247/v1)
Supplement: Supplement 1 [file 39c71c2786b63f92d82bc0bb.docx]

**
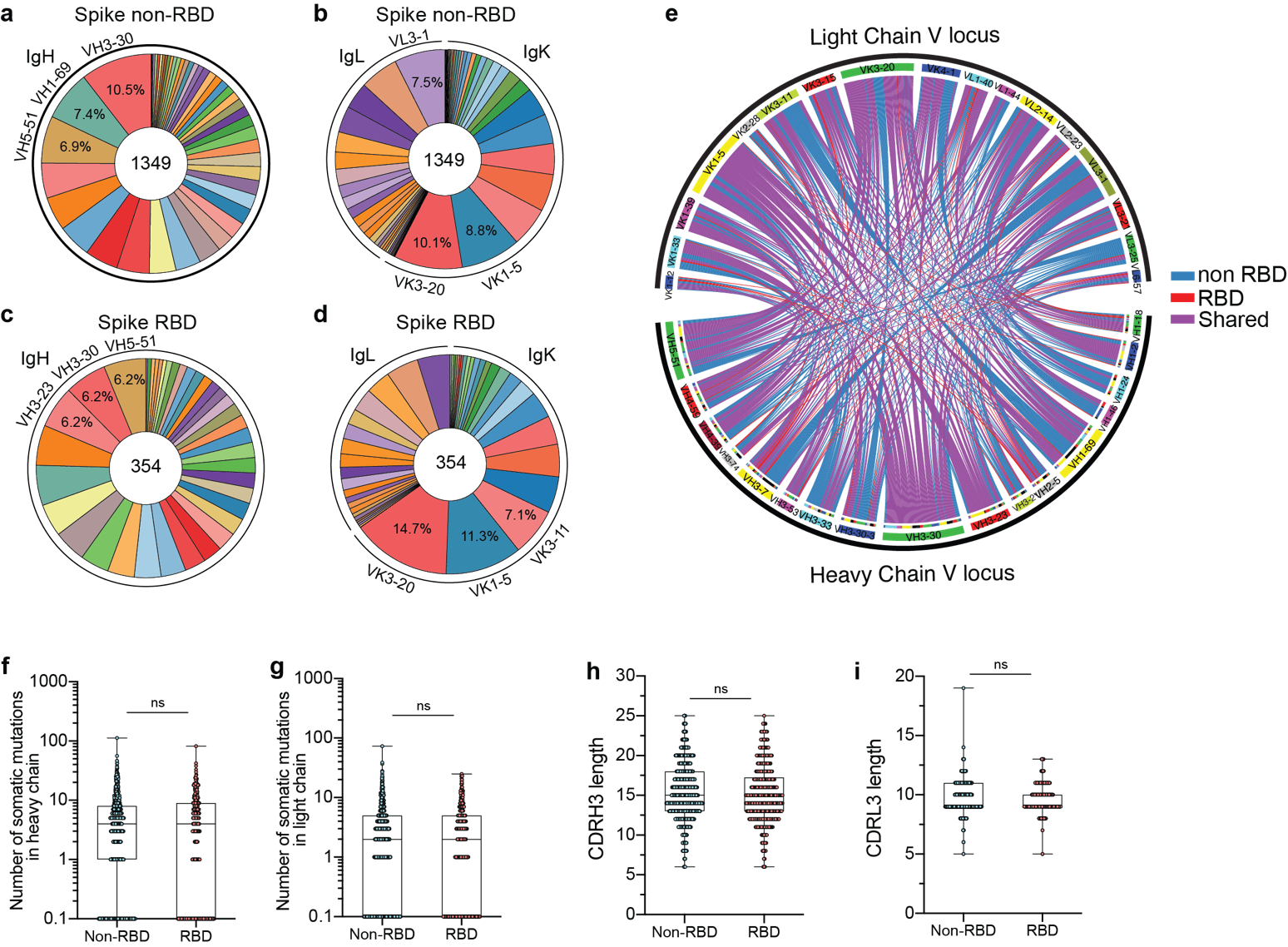
**

**Supplementary Figure 1: MAb genetic, somatic hypermutation, and CDR3 length features. a-d,** The distribution of V gene usage of spike-non RBD and spike RBD antibodies for all paired heavy **(a, c)** and light **(b, d)** chains. Percentage shown indicates proportion of the top 3 utilized genes. **e,** Clonal relationships between heavy and light chain variable gene locus of spike non-RBD and spike RBD-specific antibodies. Connecting lines represent the pairing of heavy and light chain of antibody clones specific to spike non-RBD (blue) or RBD (red) and antibody clones shared between both groups (purple). **f, g,** Comparison of number of somatic hypermutations of heavy **(f)** and light chains **(g)** of spike non-RBD and spike RBD-binding B cells. **h and i,** The complementarity determining region 3 (CDR3) amino acid length for heavy **(h)** and light chains **(i)** of spike non-RBD and spike RBD-binding B cells. Median indicated as line in the box and whisker graph. Each dot represents an individual antibody with range from minimum to maximum value. Data in **f-i** were analyzed using Mann-Whitney non-parametric test.


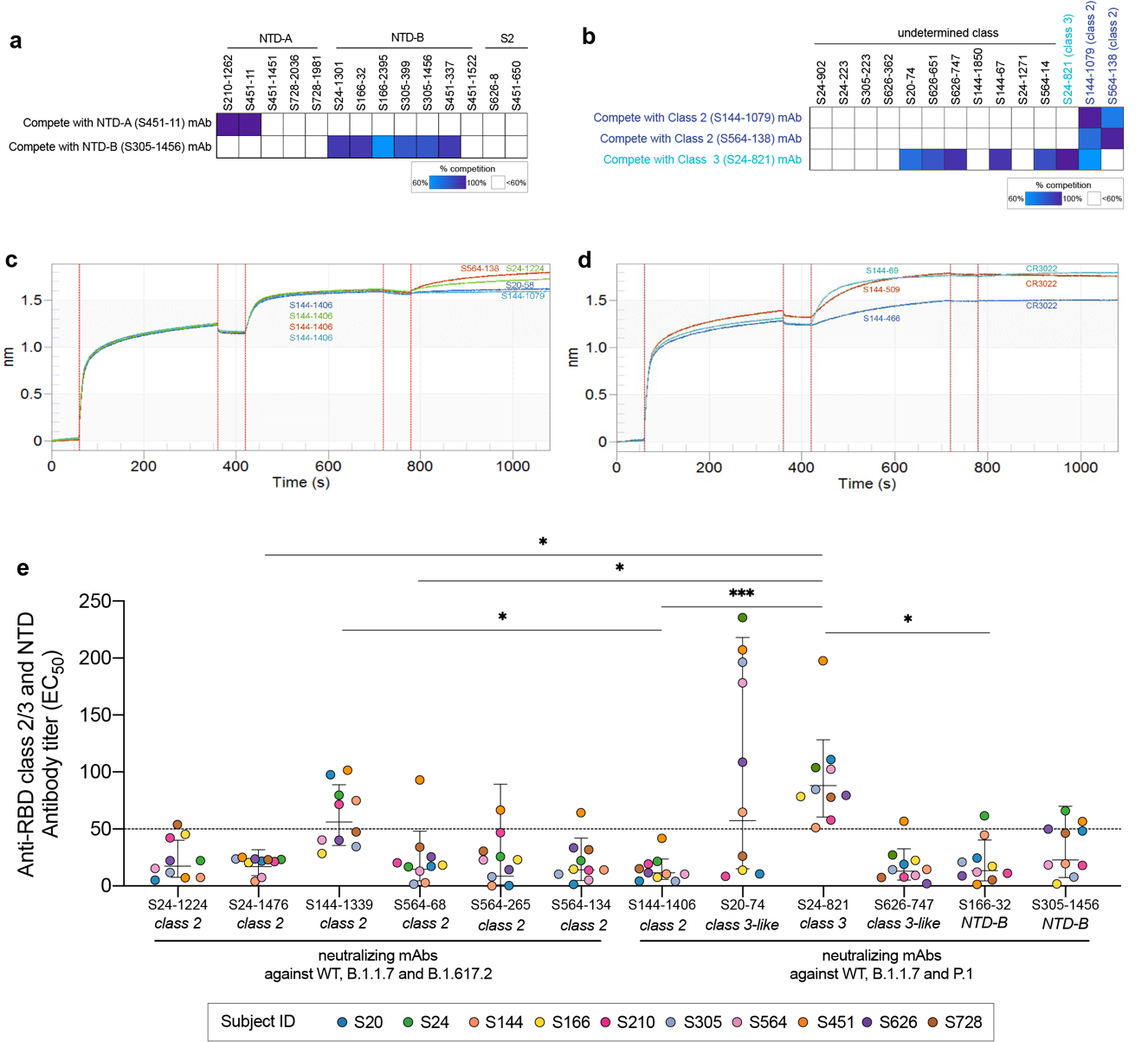


**Supplementary Figure 2: MAb binding competition by ELISA and BLI and serum competition by ELISA. a,** Competition ELISA of RBD mAbs of spike non-RBD mAbs with NTD-A (S451-11) and NTD-B (S305-1456). **b,** Competition ELISA of RBD mAbs of undetermined class with class 2 mAbs (S144-1079 and S564-138) and class 3 mAb (S24-821). **c,** MAb binding competition by BLI of class 2 mAb, S144-1406, with the other class 2 mAbs (n=4) that did not neutralize P.1. **d,** MAb binding competition by BLI between class 4 mAbs that utilized VH5-51 (S144-466, S144-509, S144 and S144-69) with CR3022. **e**, EC_50_ of serum antibodies of 10 convalescent subjects competing with RBD-reactive mAbs for binding to RBD class 2, class 3 and class 3-like epitopes, and NTD-reactive mAbs for binding to NTD-B epitopes. Dashed line represents the limit of detection**.** Data in **a**-**b** and **e** are representative of two independent experiments performed in duplicate. Data in **e** were analyzed using nonparametric Friedman's test with Dunn’s multiple comparison test.

**
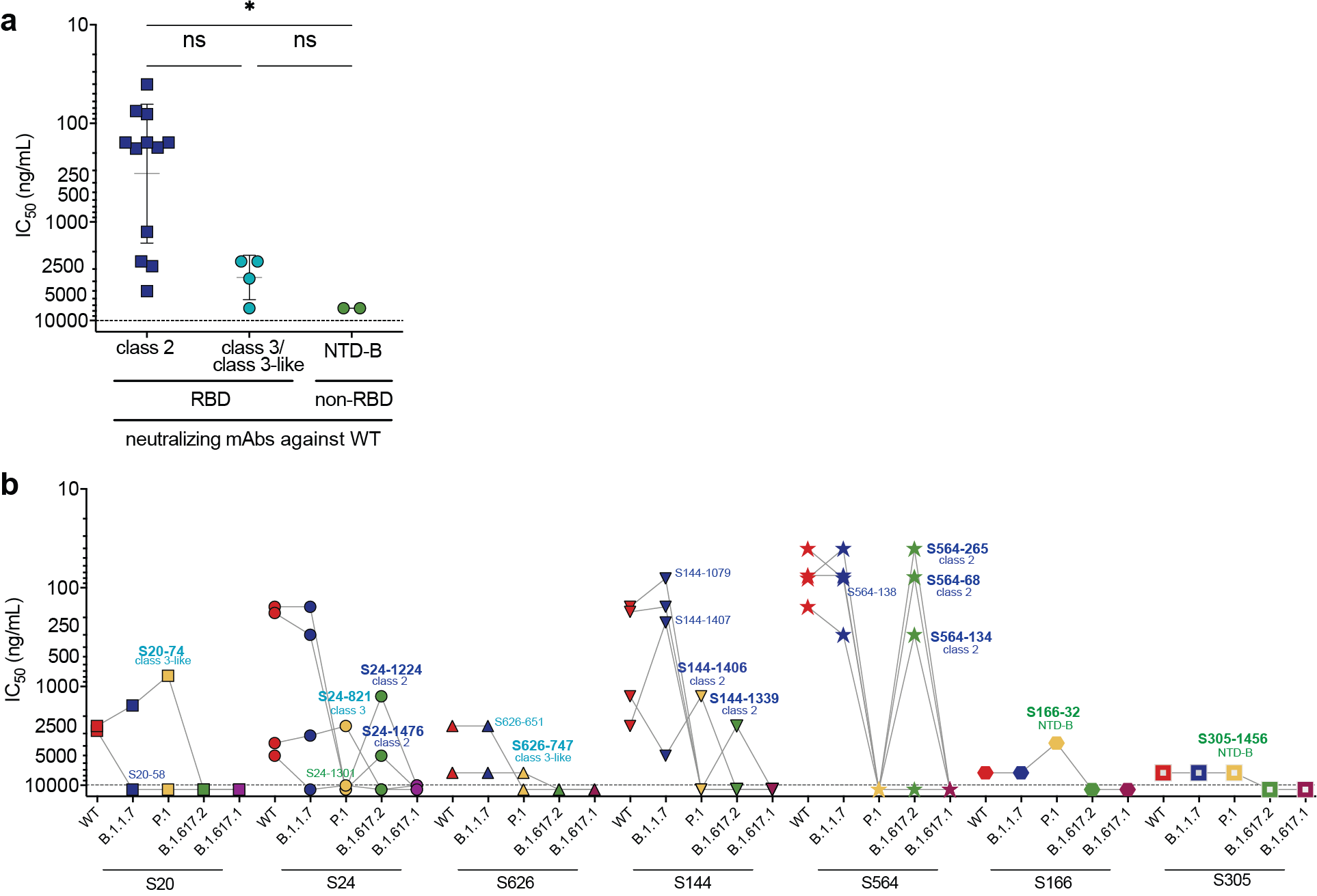
**

**Supplementary Figure 3: Comparison of neutralization potency of SARS-CoV-2 neutralizing mAbs. a,** Neutralization potency (IC_50_) of RBD-binding mAbs, class 2 and class 3, and NTD-B binding mAbs against WT SARS-CoV-2. **b,** Neutralization potency of each mAb from each subject against WT SARS-CoV-2 (red), B.1.1.7 (blue), P.1 (yellow), B.1.617.1 (green) and B.1.617.2 (plum). Each dot indicates one mAb. MAbs that neutralize VOCs are bolded. Data in **a**-**c** are representative of two independent experiments performed in duplicate. Data in **a** were analyzed using Mann-Whitney non-parametric test.

**Supplementary Table 1: COVID-19 convalescent subjects.**

| **Subject ID** | **Age** | **Sex** | **SARS-CoV-2 PCR Test** | **Duration of symptoms (days)** | **Symptom start to**  **donation (days)** |
| --- | --- | --- | --- | --- | --- |
| 24 | 34 | M | 3/23/20 | 12 | 41 |
| 20 | 31 | M | 3/31/20 | 19 | 48 |
| 564 | 24 | F | 3/19/20 | 32 | 60 |
| 144 | 56 | M | 3/16/20 | 23 | 54 |
| 305 | 43 | F | 4/17/20 | 4 | 47 |
| 166 | 42 | F | 3/25/20 | 17 | 55 |
| 210 | 47 | M | 4/4/20 | 7 | 41 |
| 451 | 46 | M | 4/4/20 | 11 | 49 |
| 626 | 44 | M | 3/31/20 | 19 | 56 |
| 728 | 62 | F | 3/15/20 | 53 | 130 |

**Supplementary Table 2: Characteristics of SARS-CoV-2 spike binding mAbs.** Cross-neutralizing mAbs against WT, B.1.1.7 and P.1 or B.1.617.2 are bolded.

| **mAb ID** | **Epitope specificity** | **VH gene** | **VL gene** | **# VH SHM** | **#VL SHM** | **CDRH3 length** | **CDRL3 length** | **Database availability** |
| --- | --- | --- | --- | --- | --- | --- | --- | --- |
| S20-58 | Spike RBD  Class 2 | IGHV4-30-4*08 | IGKV2-24*01 | 5 | 2 | 15 | 9 | reference 27 |
| **S20-74** | Spike RBD  Class 3-like | IGHV4-59*11 | IGLV2-8*01 | 7 | 3 | 15 | 11 | reference 27 |
| S24-223 | Spike RBD  undetermined | IGHV2-5*02 | IGLV2-14*01 | 1 | 3 | 11 | 11 | reference 27 |
| **S24-821** | Spike RBD  Class 3 | IGHV2-70*15 | IGKV1-5*03 | 3 | 0 | 16 | 9 | reference 27 |
| S24-902 | Spike RBD  undetermined | IGHV1-69*04 | IGLV7-46*01 | 0 | 0 | 15 | 8 | reference 27 |
| S24-1002 | Spike RBD  Class 2 | IGHV3-30-3*01 | IGKV1-13*02 | 4 | 5 | 25 | 9 | reference 27 |
| **S24-1224** | Spike RBD  Class 2 | IGHV1-46*01 | IGLV1-40*01 | 8 | 7 | 20 | 11 | reference 27 |
| S24-1271 | Spike RBD  undetermined | IGHV3-66*01 | IGLV3-1*01 | 7 | 6 | 22 | 9 | reference 27 |
| S24-1301 | Spike  NTD-B | IGHV1-24*01 | IGLV10-54*01 | 6 | 4 | 18 | 11 | reference 27 |
| S24-1384 | Spike RBD  Class 4 | IGHV3-48*04 | IGLV3-21*02 | 3 | 4 | 17 | 12 | reference 27 |
| **S24-1476** | Spike RBD  Class 2 | IGHV3-49*03 | IGKV3-15*01 | 3 | 0 | 18 | 8 | reference 27 |
| S144-67 | Spike RBD  Class 3-like | IGHV5-51*01 | IGLV1-40*01 | 8 | 5 | 17 | 12 | reference 27 |
| S144-69 | Spike RBD  Class 4 | IGHV5-51*01 | IGKV1-5*01 | 3 | 3 | 11 | 8 | reference 27 |
| S144-466 | Spike RBD  Class 4 | IGHV5-51*01 | IGKV1-5*01 | 7 | 6 | 11 | 9 | reference 27 |
| S144-509 | Spike RBD  Class 4 | IGHV5-51*01 | IGKV1-5*01 | 3 | 1 | 12 | 9 | reference 27 |
| S144-1079 | Spike RBD  Class 2 | IGHV1-69*02 | IGKV3-20*01 | 9 | 3 | 19 | 9 | reference 27 |
| **S144-1339** | Spike RBD  Class 2 | IGHV1-2*06 | IGLV2-14*01 | 15 | 5 | 18 | 11 | reference 27 |
| **S144-1406** | Spike RBD  Class 2 | IGHV1-3*01 | IGKV1-5*01 | 4 | 0 | 11 | 17 | reference 27 |
| S144-1407 | Spike RBD  Class 2 | IGHV1-69*02 | IGKV1-5*01 | 9 | 2 | 12 | 10 | reference 27 |
| S144-1850 | Spike RBD  undetermined | IGHV3-23*04 | IGKV1-5*01 | 2 | 3 | 13 | 9 | reference 27 |
| **S166-32** | Spike  NTD-B | IGHV3-11*01 | IGKV1-5*01 | 10 | 2 | 19 | 8 | reference 27 |
| S166-2395 | Spike  NTD-B | IGHV4-4*07 | IGLV3-21*02 | 3 | 5 | 16 | 12 | reference 27 |
| S210-1262 | Spike  NTD-A | IGHV4-39*01 | IGLV4-69*01 | 11 | 4 | 10 | 9 | reference 27 |
| S305-223 | Spike RBD  Class 2 | IGHV3-33*06 | IGKV3-11*01 | 18 | 8 | 6 | 9 | reference 27 |
| S305-399 | Spike RBD  undetermined | IGHV1-24*01 | IGKV3-15*01 | 4 | 4 | 18 | 9 | reference 27 |
| **S305-1456** | Spike  NTD-B | IGHV1-24*01 | IGKV3-15*01 | 3 | 3 | 20 | 9 | reference 27 |
| S451-11 | Spike  NTD-A | IGHV3-23*01 | IGKV3D-20*01 | 8 | 3 | 15 | 8 | internal data |
| S451-337 | Spike  NTD-B | IGHV4-59*01 | IGKV3-20*01 | 10 | 1 | 15 | 10 | internal data |
| S451-650 | Spike S2 | IGHV3-30*01 | IGKV3-20*01 | 6 | 4 | 14 | 8 | internal data |
| S451-1451 | Spike  NTD-A | IGHV4-31*01 | IGLV2-11*01 | 9 | 5 | 14 | 10 | internal data |
| S451-1522 | Spike  NTD-B | IGHV2-26*01 | IGLV2-14*01 | 8 | 5 | 20 | 11 | internal data |
| S564-14 | Spike RBD  Class 3-like | IGHV3-7*01 | IGLV3-21*04 | 6 | 3 | 18 | 12 | reference 27 |
| **S564-68** | Spike RBD  Class 2 | IGHV1-2*02 | IGLV2-8*01 | 6 | 2 | 15 | 10 | reference 27 |
| **S564-134** | Spike RBD  Class 2 | IGHV1-2*02 | IGLV2-8*01 | 2 | 6 | 15 | 10 | reference 27 |
| S564-138 | Spike RBD  Class 2 | IGHV1-2*02 | IGLV2-14*01 | 10 | 1 | 18 | 10 | reference 27 |
| S564-152 | Spike RBD  Class 4 | IGHV3-33*06 | IGKV1-33*01 | 4 | 4 | 20 | 10 | reference 27 |
| **S564-265** | Spike RBD  Class 2 | IGHV1-2*02 | IGLV2-8*01 | 4 | 3 | 15 | 10 | reference 27 |
| S626-8 | Spike S2 | IGHV1-8*01 | IGLV3-19*01 | 7 | 5 | 24 | 12 | this study |
| S626-362 | Spike RBD  undetermined | IGHV3-48*01 | IGLV1-40*01 | 18 | 3 | 16 | 11 | this study |
| S626-651 | Spike RBD  Class 3-like | IGHV1-69*04 | IGLV1-40*01 | 6 | 4 | 17 | 11 | this study |
| **S626-747** | Spike RBD  Class 3-like | IGHV3-9*01 | IGKV1-33*01 | 6 | 6 | 22 | 10 | this study |
| S728-1981 | Spike  NTD-A | IGHV1-46*01 | IGKV3-11*01 | 17 | 3 | 16 | 11 | this study |
| S728-2036 | Spike  NTD-A | IGHV1-2*02 | IGLV2-23*02 | 14 | 12 | 17 | 10 | this study |

**Supplementary Table 3: Antigen information and source.** VOC refers to variant of concern and VOI refers to variant of interest.

| **Antigen** | **S1 NTD** | **RBD** | **S1 CTD** | **S2** | **Mutation detected in** | **Source** |
| --- | --- | --- | --- | --- | --- | --- |
| Spike FL, 2-P, trimer | | | | | |  |
| WT | - | - | - | - | - | In-house |
| D614G | - | - | D614G | - | VOC | In-house |
| B.1.1.7 | H69del, V70del, Y144del | N501Y | A570D, D614G, P681H | T716I, S982A, D1118H | VOC | Sather lab |
| B.1.351 | L18F, D80A, D215G, del241-243, R246I | K417N, E484K, N501Y | D614G | A701V | VOC | Sather lab |
| P.1 | L18F, T20N, P26S, D138Y, R190S | K417T, E484K, N501Y | D614G, H655Y | T1027I, V1176F | VOC | Sather lab |
| B.1.526 | L5F, T95I, D253G | E484K | D614G | A701V | VOI | Sather lab |
| B.1.617.1 | T95I, G142D, E154K | L452R, E484Q | D614G, P681R | Q1071H | VOI | Sather lab |
| A.23.1 | F157L | V367F | Q613H, P681R | - | VOI | Sather lab |
| S1 monomeric | | | | | |  |
| WT | - | - | - | - | - | SinoBiological |
| S2 monomeric | | | | | |  |
| WT | - | - | - | - | - | SinoBiological |
| RBD | | | | | |  |
| WT | - | - | - | - | - | In-house |
| E406Q | - | E406Q | - | - | Circulating variant*, In vitro* escape | Krammer lab |
| K417N (B.1.351) | - | K417N | - | - | VOC, *In vitro* escape | SinoBiological |
| K417T (P.1) | - | K417T | - | - | VOC, *In vitro* escape | In-house |
| K417E | - | K417E | - | - | *In vitro* escape | Krammer lab |
| K417V | - | K417V | - | - | *In vitro* escape | Krammer lab |
| K417A | - | K417A | - | - | RBD-ACE2 contacting | In-house |
| Y453F (B.1.427, B.1.429) | - | Y453F | - | - | VOC, *In vitro* escape | Krammer lab |
| F486A | - | F486A | - | - | *In vitro* escape | Krammer lab |
| N487R | - | N487R | - | - | *In vitro* escape | Krammer lab |
| E484K (P.1, B.1.526, B.1.351, B.1.1.318, B.1.525, R.1, B.1.526.2, B.1.1, B.1.621, B.1, B.1.1.7) | - | E484K | - | - | VOC, *In vitro* escape | Krammer lab |
| F490K | - | F490K | - | - | *In vitro* escape | Krammer lab |
| Q493R | - | Q493R | - | - | *In vitro* escape | Krammer lab |
| N439K | - | N439K | - | - | VOC, *In vitro* escape | Krammer lab |
| N440K (B.1.36) | - | N440K | - | - | Circulating variant, *In vitro* escape | Krammer lab |
| L452R (B.1.526.1, B.1.429, B.1.427, B.1.617.2, B.1, B.1.617.1, C.36, A.2.5) | - | L452R | - | - | VOC, *In vitro* escape | SinoBiological |
| N501Y (B.1.1.7) | - | N501Y | - | - | VOC | Krammer lab |
| N501Q | - | N501Q | - | - | RBD-ACE2 contacting | Krammer lab |
| N501A | - | N501A | - | - | RBD-ACE2 contacting | Krammer lab |
| B.1.351 |  | K417N, E484K, N501Y |  |  | VOC | Krammer lab |
| P.1 |  | K417T, E484K, N501Y |  |  | VOC | Krammer lab |
| Other coronaviruses | | | | | |  |
| SARS-CoV-1 RBD WT | | | | | - | In-house |
| MERS-CoV RBD WT | | | | | - | In-house |

**Supplementary Table 4: SARS-CoV-2 virus information and source.**

| **Antigen** | **S1 NTD** | **RBD** | **S1 CTD** | **S2** | **Source** |
| --- | --- | --- | --- | --- | --- |
| WT | - | - | - | - | SARS-CoV-2/UT-NCGM02/Human/2020/Tokyo from BEI |
| B.1.1.7 | L5F, H69del, V70del, Y144del | N501Y | A570D, D614G, P681H | T716I, S982A, D1118H | hCoV-19/Japan/QHN001/2020 from BEI |
| P.1 | L18F, T20N, P26S, D138Y, G181V, R190S | K417T, E484K, N501Y | D614G, H655Y | T1027I, V1176F | hCoV-19/Japan/TY7-501/2021 from BEI |
| B.1.617.1 | G142D, E154K | L452R, E484Q | D614G, P681R | Q1071H, H1101D | hCoV-19/USA/CA-Stanford-15_S02/2021 from BEI |
| B.1.617.2 | T19R, T95I, G142D, E156G, F157del, R158del | L452R, T478K | D614G, P681R | D950N | hCoV-19/USA/WI-UW-5250/2021 |
