# Supplementary material for: Cross neutralization of emerging SARS-CoV-2 variants of concern by antibodies targeting distinct epitopes on spike
Source: Res Sq. 2021 Jul 19:rs.3.rs-678247. Preprint. [Version 1] doi: 10.21203/rs.3.rs-678247/v1 (PMC8312900; doi:10.21203/rs.3.rs-678247/v1)
Supplement: Supplement 2 [file b6454d8f9809f4dbaa025d75.pdf]

nature portfolio

Corresponding author(s): Patrick C. Wilson

Last updated by author(s): Jul. 14, 2021

Reporting Summary

Nature Portfolio wishes to improve the reproducibility of the work that we publish. This form provides structure for consistency and transparency in reporting. For further information on Nature Portfolio policies, see our Editorial Policies and the Editorial Policy Checklist.

Statistics

For all statistical analyses, confirm that the following items are present in the figure legend, table legend, main text, or Methods section.

n/a	Confirmed	
☐	☒	The exact sample size (*n*) for each experimental group/condition, given as a discrete number and unit of measurement
☐	☒	A statement on whether measurements were taken from distinct samples or whether the same sample was measured repeatedly
☐	☒	The statistical test(s) used AND whether they are one- or two-sided<br>*Only common tests should be described solely by name; describe more complex techniques in the Methods section.*
☒	☐	A description of all covariates tested
☐	☒	A description of any assumptions or corrections, such as tests of normality and adjustment for multiple comparisons
☐	☒	A full description of the statistical parameters including central tendency (e.g. means) or other basic estimates (e.g. regression coefficient) AND variation (e.g. standard deviation) or associated estimates of uncertainty (e.g. confidence intervals)
☐	☒	For null hypothesis testing, the test statistic (e.g. *F*, *t*, *r*) with confidence intervals, effect sizes, degrees of freedom and *P* value noted<br>*Give P values as exact values whenever suitable.*
☒	☐	For Bayesian analysis, information on the choice of priors and Markov chain Monte Carlo settings
☒	☐	For hierarchical and complex designs, identification of the appropriate level for tests and full reporting of outcomes
☒	☐	Estimates of effect sizes (e.g. Cohen's *d*, Pearson's *r*), indicating how they were calculated

Our web collection on statistics for biologists contains articles on many of the points above.

Software and code

Policy information about availability of computer code

Data collection	MACSQuantifyTyto Software 0.5 for cell sorting; Illumina NextSeq 550/500; Microplate Manager® 6, Version 6.3 for ELISA data collection
Data analysis	FlowJo 10.7.1 for FACS analysis; Cell Ranger 3.0.2 for 10 × Genomics; IgBlast; GraphPad Prism V_9.0.1; IgBlast; IMGT/V-QUEST; LinQ-View 0.9.9 (https://wilsonimmunologylab.github.io/LinQView/); Adobe Illustrator 2021; Microsoft Word/Excel; Seurat v3.9.9 (https://satijalab.org/seurat/); Umap v0.2.6.0 (https://github.com/lmcinnes/umap)

For manuscripts utilizing custom algorithms or software that are central to the research but not yet described in published literature, software must be made available to editors and reviewers. We strongly encourage code deposition in a community repository (e.g. GitHub). See the Nature Portfolio guidelines for submitting code & software for further information.

Data

Policy information about availability of data

All manuscripts must include a data availability statement. This statement should provide the following information, where applicable:

- Accession codes, unique identifiers, or web links for publicly available datasets
- A description of any restrictions on data availability
- For clinical datasets or third party data, please ensure that the statement adheres to our policy

Details about the monoclonal antibodies, recombinant antigens and live virus are provided in SI Table 1-4. The raw sequencing data and scripts for the single cell RNA sequencing are available by visiting https://data.mendeley.com/datasets/3jdywv5jrv/3. The expression vectors this study used for antibody production can be referred from genbank (FJ475055, FJ475056, and FJ517647). Some data are from published research 'Profiling B cell immunodominance after SARS-CoV-2 infection reveals antibody evolution to non-neutralizing viral targets'. (doi: 10.1016/j.immuni.2021.05.001.)

Field-specific reporting

Please select the one below that is the best fit for your research. If you are not sure, read the appropriate sections before making your selection.

☒ Life sciences ☐ Behavioural & social sciences ☐ Ecological, evolutionary & environmental sciences

For a reference copy of the document with all sections, see nature.com/documents/nr-reporting-summary-flat.pdf

Life sciences study design

All studies must disclose on these points even when the disclosure is negative.

Sample size	Sample size = 10
Data exclusions	No data were excluded.
Replication	All experiments were performed at least twice.
Randomization	It is not relevant as it is an observational study.
Blinding	It is not relevant as it is an observational study.

Reporting for specific materials, systems and methods

We require information from authors about some types of materials, experimental systems and methods used in many studies. Here, indicate whether each material, system or method listed is relevant to your study. If you are not sure if a list item applies to your research, read the appropriate section before selecting a response.

Materials & experimental systems

n/a	Involved in the study	
☐	☒	Antibodies
☐	☒	Eukaryotic cell lines
☒	☐	Palaeontology and archaeology
☒	☐	Animals and other organisms
☐	☒	Human research participants
☐	☒	Clinical data
☒	☐	Dual use research of concern

Methods

n/a	Involved in the study	
☒	☐	ChIP-seq
☐	☒	Flow cytometry
☒	☐	MRI-based neuroimaging

Antibodies

Antibodies used	PE/Cy7 anti-Human CD19 (Clone: HIB19) Biolegend Cat# 302215 - Lot# B242978;<br>Brilliant Violet 421 anti-human CD27(Clone: O323) Biolegend Cat# 302823 - Lot# B255089;<br>BV510 Mouse Anti-Human CD3 (Clone UCHT1)BD Biosciences Cat#563109;<br>BB515 Mouse Anti-Human CD38 (Clone: HIT2)BD Biosciences Cat# 564499 - Lot# 9353306;<br>Goat anti-human IgG (Fab-specific) HRP Sigma Cat# A0293;<br>All the primary antibody used in ELISA assay were produced in-house.
Validation	No validation for commercial antibodies.

Eukaryotic cell lines

Policy information about cell lines

Cell line source(s)	HEK293T cells were obtained from ATCC (CRL-11268). Expi293F cells were purchased from ThermoFisher Corporation.
Authentication	Not authenticated.
Mycoplasma contamination	The cells were tested negative for mycoplasm contamination.
Commonly misidentified lines (See ICLAC register)	No commonly misidentified cell lines were used.

Human research participants

Policy information about studies involving human research participants

Population characteristics	Informed consent was obtained after the research applications and possible consequences of the studies were disclosed to study subjects. This clinical trial was registered at ClinicalTrials.gov with identifier NCT04340050, and clinical information for patients included in the study is detailed in Tables S1. Convalescent leukoreduction filter donors were 18 years of age or older, eligible to donate blood as per standard University of Chicago Medicine Blood Donation Center guidelines, had a documented COVID-19 polymerase chain reaction (PCR) positive test, and complete resolution of symptoms at least 28 days prior to donation. Severe acute infected blood donors were 18 years of age or older and blood was collected per standard University of Chicago Medical Center guidelines. Subjects had a documented COVID-19 polymerase chain reaction (PCR) positive test, were hospitalized, and had been scheduled to receive an infusion of convalescent donor plasma. Four blood draws were collected both before and after plasma infusion, at days 0, 1, 3, and 14.
Recruitment	The participants were recruited at no biases. The ages of subjects were from 24 to 62; the duration of symptoms is ranging from 4 to 53 days. The days of symptom start to donation are from 41 to 130.
Ethics oversight	All studies were performed with the approval of the University of Chicago institutional review board IRB20-0523 and University of Chicago, University of Wisconsin-Madison, and Washington University in St. Louis institutional biosafety committees.

Note that full information on the approval of the study protocol must also be provided in the manuscript.

Clinical data

Policy information about clinical studies

All manuscripts should comply with the ICMJE guidelines for publication of clinical research and a completed CONSORT checklist must be included with all submissions.

Clinical trial registration	NCT04340050
Study protocol	All studies were performed with the approval of the University of Chicago institutional review board IRB20-0523.
Data collection	the samples were collected at the University of Chicago Medicine in April, May and July of 2020.
Outcomes	The primary outcome is that neutralizing antibodies against current variants do exist in the sera of patients with a history of natural infection and the secondary outcome is that it is critical to induce antibodies targeting multiple distinct epitopes of the spike that can neutralize emerging variants of concern.

Flow Cytometry

Plots

Confirm that:

- [x] The axis labels state the marker and fluorochrome used (e.g. CD4-FITC).
- [x] The axis scales are clearly visible. Include numbers along axes only for bottom left plot of group (a 'group' is an analysis of identical markers).
- [x] All plots are contour plots with outliers or pseudocolor plots.
- [x] A numerical value for number of cells or percentage (with statistics) is provided.

Methodology

Sample preparation	PBMCs were collected from leukoreduction filters or blood draws within 2 hours post-collection and, if applicable, flushed from the filters using sterile 1X Phosphate-Buffered Saline supplemented with 0.2% Bovine Serum Albumin Lymphocytes were purified by Lymphoprep Ficoll gradient and contaminating red blood cells were lysed by ACK buffer. Cells were frozen in Fetal Bovine Serum with 10% Dimethyl sulfoxide prior to downstream analysis. On the day of sorting, B cells were enriched using the human pan B cell EasySepTM enrichment kit.
Instrument	MACSQuantTyto cartridge sorting platform (Miltenyi)
Software	MACSQuantifyTyto Software0.5
Cell population abundance	The percentage of antigen-specific B cells was ranging from 0.02-1.25 among enriched peripheral B cells.
Gating strategy	Cells were sorted as viable/CD19+/antigen-PE+ or viable/CD19+/antigen-APC+. The PE+ and APC+ gates were drawn by use of FMO controls.

- [ ] Tick this box to confirm that a figure exemplifying the gating strategy is provided in the Supplementary Information.
